# A systematic review and meta-analysis of observational studies on the effects of epigenetic factors on serum triglycerides

**DOI:** 10.20945/2359-3997000000472

**Published:** 2022-05-12

**Authors:** Sadegh Mazaheri-Tehrani, Mehri Khoshhali, Motahar Heidari-Beni, Parnian Poursafa, Roya Kelishadi

**Affiliations:** 1 Isfahan University of Medical Sciences Research Institute for Primordial Prevention of Non-Communicable Disease Child Growth and Development Research Center Isfahan Iran MD student, Child Growth and Development Research Center, Research Institute for Primordial Prevention of Non-Communicable Disease, Isfahan University of Medical Sciences, Isfahan, Iran; 2 Isfahan University of Medical Sciences Research Institute for Primordial Prevention of Non-communicable Disease Department of Pediatrics, Child Growth and Development Research Center Isfahan Iran PhD of Biostatistics. Department of Pediatrics, Child Growth and Development Research Center, Research Institute for Primordial Prevention of Non-communicable Disease, Isfahan University of Medical Sciences, Isfahan, Iran; 3 Isfahan University of Medical Sciences Research Institute for Primordial Prevention of Non-Communicable Disease Department of Nutrition Isfahan Iran Assistant Professor, Department of Nutrition, Child Growth and Development Research Center, Research Institute for Primordial Prevention of Non-Communicable Disease, Isfahan University of Medical Sciences, Isfahan, Iran; 4 University of Isfahan Faculty of Science Department of Cellular and Molecular Biology Isfahan Iran MSc Student, Department of Cellular and Molecular Biology, Faculty of Science, University of Isfahan, Isfahan, Iran; 5 Isfahan University of Medical Sciences Research Institute for Primordial Prevention of Non-Communicable Disease Department of Pediatrics Isfahan Iran Professor, Department of Pediatrics, Child Growth and Development Research Center, Research Institute for Primordial Prevention of Non-Communicable Disease, Isfahan University of Medical Sciences, Isfahan, Iran

**Keywords:** Triglycerides, epigenomics, DNA methylation, meta-analysis

## Abstract

Epigenetic modifications might be associated with serum triglycerides (TG) levels. This study aims to systematically review the studies on the relationship between the methylation of specific cytosine-phosphate-guanine (CpG) sites and serum TG levels. This systematic review and meta-analysis study was conducted according to the PRISMA 2020 (Preferred Reporting Items for Systematic Reviews and Meta-Analyses) statement. A systematic literature search was conducted in Medline database (PubMed), Scopus, and Cochrane library up to end of 2020. All observational studies (cross-sectional, case-control, and cohort) were included. Studies that assessed the effect of DNA methylation of different CpG sites of *ABCG1, CPT1A,* and *SREBF1* genes on serum TG levels were selected. The National Institutes of Health (NIH) checklist was used to assess the quality of included articles. Among 2790 articles, ten studies were included in the quantitative analysis and fourteen studies were included in the systematic review. DNA methylation of *ABCG1* gene had significant positive association with TG levels (β = 0.05, 95% CI = 0.04, 0.05, P heterogeneity < 0.001). There was significant inverse association between DNA methylation of CPT1A gene and serum TG levels (β = −0.03, 95% CI = −0.03, −0.02, P heterogeneity < 0.001). DNA methylation of *SREBF1* gene was positively and significantly associated with serum TG levels (β = 0.03; 95% CI = 0.02-0.04, P heterogeneity < 0.001). DNA methylation of *ABCG1* and *SREBF1* genes has positive association with serum TG level, whereas this association is opposite for CPT1A gene. The role of epigenetic factors should be considered in some populations with high prevalence of hypertriglyceridemia.

## INTRODUCTION

Elevated level of serum triglycerides (TG) is one of the major components of the metabolic syndrome. It is associated with adverse health effects including cardiovascular disease (CVD), obesity, and insulin resistance (1–3). Some evidence has recommended that hypertriglyceridemia should be managed in any severity (1,4). The prevalence of hypertriglyceridemia has large variations in different population and is highly influenced by different variables such as gender and age (5,6). Approximately one fifth of adult females and about one third of adult males have hypertriglyceridemia in Spain (6). A cross-sectional study reported that the mean TG level has decreased from 2007 to 2014 in the US (7). Based on cross-sectional studies in Indian population over 20-year period, prevalence of hypertriglyceridemia has increased from 25.7% to 32.8% (8). Some factors including lifestyle, environmental and genetic factors influence on serum lipid profiles (9). The role of epigenetic factors needs to be clarified in this regard.

Epigenetic processes are natural and require for functions of various organisms. However, their improper occurrence will have detrimental impacts on health and behavioral. Epigenetic changes are DNA changes without any effect on DNA sequences but impact on gene expression and activity (9,10). Several types of epigenetic processes have been identified that DNA methylation is one of the most important epigenetic changes. The covalent transfer of a methyl group to cytosine-guanine (CpG) dinucleotides of DNA by DNA methyltransferase leads to DNA methylation (11,12). CpG dinucleotides are often located in regulatory parts of genes, so they can regulate genes expression (13).

Several studies have assessed the relationship between DNA methylation at different loci of different genes with serum TG levels, and had inconsistent findings. Some results did not show significant association between DNA methylation and TG levels (11,13), whereas some of them confirmed this association (1,9,14–16). Different sample size, gene, method, and design might explain inconsistent findings. There are several epigenetic changes that might affect TG levels; some genes including *ABCG1*, *CPT1A* and *SREBF1* have been assessed more than others. The contradictory findings of various studies show the importance of providing a holistic overview. Therefore, this systematic review and meta-analysis aims to provide a summary of the literature that have evaluated the relationship between methylation of different CpGs of *ABCG1*, *CPT1A*, *SREBF1* genes and serum TG levels.

## METHODS

### Search strategy

The current systematic review and meta-analysis study was conducted according to the PRISMA 2020 (Preferred Reporting Items for Systematic Reviews and Meta-Analyses) statement (17). The protocol was registered on PROSPERO (ID: CRD42020202332). A systematic literature search was conducted in Medline database (PubMed), Scopus, and Cochrane library up to end of July 2020. Databases were searched daily for any newly published articles up to end of December 2020, and updated till September 2021. The following search terms were used: (Triglyceride OR TG OR Triacylglycerol OR Hypertriglyceridemia) AND (Epigenetic OR Epigenomic OR methylation OR “DNA methylation” OR acetylation OR “DNA acetylation”). The search string for each database was summarized in the supplementary file 1.

Two reviewers (SMT and MHB) independently reviewed and screened the appropriate published papers based on title, abstract, and full text. In addition, the reference lists of related review articles were checked to find undetected relevant studies. Any discrepancy related to eligible records was resolved by the third reviewer (RK).

### Inclusion criteria

Only English-language articles and human studies were included. We included studies that extracted DNA from blood samples and measured DNA methylation. All observational studies (cross-sectional, case-control, and cohort) on individuals over 18 years of age that assessed the effect of epigenetic changes on serum TG levels were included without restriction of gender, race, ethnicity, and year of publication.

### Exclusion criteria

Those papers with the following criteria were excluded: duplicate publications and studies that assessed the effect of hypertriglyceridemia on epigenetic changes.

### Data extraction

The number of studies on some genes including *ABCG1*, *CPT1A*, and *SREBF1* were greater than others. So, we selected them for the meta-analysis. Data extraction was conducted by two reviewers (SMT and MHB) independently and was checked by the third reviewer (RK). The following information was extracted from eligible studies: first author, publication year, study design, sample size, age, body mass index (BMI), the gene(s) and loci and type of tissue sample.

### Quality assessment

In order to evaluate the risk of bias of 14 included studies in the systematic review, two reviewers (SMT, MHB) independently assessed the quality of the articles by National Institutes of Health (NIH) Quality Assessment Tool (18). All included studies had cohort and cross-sectional design. Any disagreement was resolved by consulting with the third researcher (RK). The scale consist of 14 questions. Each item was answered as “yes”, “no”, “not applicable” or “not reported”. Table 1 shows the quality assessment of included articles.

**Tabla 1:** Quality assessment of included studies by The National Institutes of Health (NIH) Quality Assessment Tool

Author, year	1	2	3	4	5	6	7	8	9	10	11	12	13	14	Quality Rating
Truong et al. (2017) (1)	Yes	Yes	Yes	Yes	Yes	No	No	NA	Yes	No	Yes	NA	NA	Yes	Good
Braun et al. (2017) (12)	Yes	Yes	Yes	Yes	No	Yes	No	NA	Yes	Yes	Yes	NA	No	Yes	Good
Campanella et al. (2018) (23)	Yes	Yes	Yes	Yes	No	Yes	No	NA	Yes	Yes	Yes	NA	No	Yes	Good
Dayeh et al. (2016) (19)	Yes	Yes	Yes	Yes	No	No	No	NA	No	No	Yes	NA	NA	Yes	Fair
Pfeiffer et al. (2015) (15)	Yes	Yes	Yes	Yes	No	Yes	No	NA	Yes	Yes	Yes	NA	No	Yes	Good
Guay et al. (2016) (11)	Yes	Yes	No	Yes	No	No	No	NA	Yes	No	No	NA	No	Yes	Fair
Peng et al. (2014) (13)	Yes	Yes	Yes	Yes	No	No	No	NA	Yes	No	Yes	NA	NA	Yes	Good
Wei & Wu (2018) (10)	Yes	Yes	Yes	Yes	No	No	No	NA	Yes	No	Yes	NA	NA	Yes	Good
Sayols-Baixeras et al. (2016) (9)	Yes	Yes	Yes	Yes	No	No	No	NA	Yes	Yes	Yes	NA	No	Yes	Good
Irvin et al. (2014) (16)	Yes	Yes	Yes	Yes	No	No	No	NA	Yes	Yes	Yes	NA	No	Yes	Good
Romanescu et al. (2018) (30)	Yes	Yes	Yes	Yes	No	No	No	NA	Yes	No	Yes	NA	NA	Yes	Good
Hedman et al. (2017) (14)	Yes	Yes	Yes	Yes	No	No	No	NA	Yes	Yes	Yes	NA	No	Yes	Good
Gagnon et al. (2014) (31)	Yes	Yes	No	Yes	No	No	No	NA	Yes	Yes	Yes	NA	No	Yes	Fair
Lai et al. (2016) (24)	Yes	Yes	Yes	Yes	No	No	No	NA	Yes	Yes	Yes	NA	No	Yes	Good

### Statistical analysis

The β-coefficient values of selected studies were applied for pooled analysis. The potential heterogeneity across studies was evaluated using the Cochran's Q-test and was expressed using the I^2^ index. The pooled results were calculated by the random-effects model.

Subgroup analyses based on CpG sites were performed to seek the sources of heterogeneity. In addition, meta-regression was used for assessing the mean age, mean BMI, sample size and the year of publication of studies as the possible source of heterogeneity. The sensitivity analyses were performed by excluding one study at a time to gauge the robustness of our results. Publication bias was evaluated by Funnel plot and Egger's test. The possible publication bias was adjusted using the trim and fill method. Statistical analyses were conducted using the STATA 12.0 software (STATA Corp, College Station, Texas, USA). P < 0.05 was considered as significance level.

## RESULTS

### Study selection

**Figure 1:**
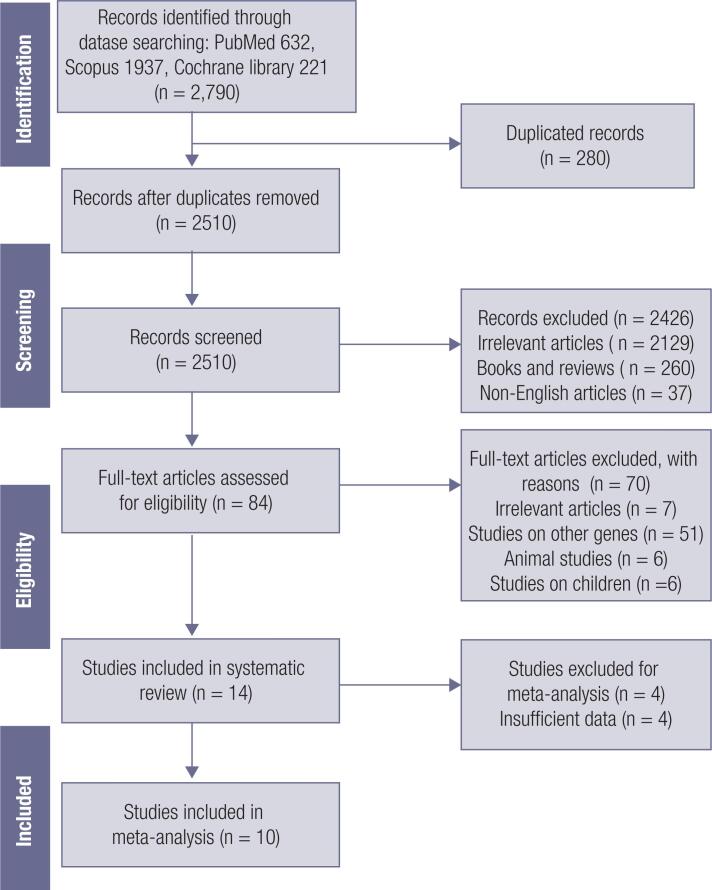
Literature search for the meta-analysis.

The flow diagram for the process of study selection is shown in Figure 1. The initial search recognized 2,790 articles and 2510 of them remained after excluding duplicates. After screening the title and abstracts, 2,426 articles were excluded, and 84 articles remained for further assessment. The full texts of remaining studies were reviewed carefully by two researchers. Any discrepancy was resolved by the third reviewer. Finally, 14 articles were included in the systematic review, and 10 of them were included in the meta-analysis.

### Study characteristics

Fourteen eligible studies were included in the systematic review. Table 2 shows the general characteristics of the included studies.

**Tabla 2:** Characteristics of included studies according to gene methylation sites and serum triglycerides level

Author & year	Country	Study design	Study characteristics				Gene & Methylation sites^2^	Adjustment	Main findings	QR^3^
			Study name	Population (male%)	Age^1^	BMI^1^				
Truong et al. (2017) (1)	Canada	Cross-sectional	Discovery set	n = 199 (47%)	39.6 ± 16.9	26.8 ± 6.1	1	Age, sex, and cell type proportions	Positive association	Good
			Replication set	n = 324 (22%)	44.1 ± 14.3	24.2 ± 4.4				
Braun et al. (2017) (12)	Netherland	Cohort	Discovery set	n = 725 (46%)	59.9 ± 8.2	27.6 ± 4.6	1, 7, 8, 12	Age, sex, current smoking, leukocyte proportions, lipid-lowering medication use, adjusted for waist circumference and other serum lipids	Negative association for *CPT1A* and positive association for *SREBF1* & *ABCG1*	Good
			Replication set	n = 760 (42%)	67.7 ± 5.9	27.8 ± 4.2				
Campanella et al. (2018) (23)	USA	Pooled analysis of Cohorts	NA	n = 1941 (30%)	NA	NA	1, 2, 7, 12	Sex, age, and status such as cancers, MI, BMI & WHR	Negative association for *CPT1A* and positive association for *SREBF1* & *ABCG1*	Good
Dayeh et al. (2016) (19)	Finland	Cohort	NA	n = 209	NA	NA	1	Age, gender, fasting glucose, and family relation	Positive association	Fair
Pfeiffer et al. (2015) (15)	Germany	Cohort	Discovery set (KORA F4 study)	n = 1776 (49%)	60.8 ± 8.9	28.2 ± 4.8	1, 2, 3, 7, 12, 13	Age, sex, BMI, smoking, alcohol, lipid-lowering drugs, physical activity, history of MI, current hypertension, HbA1c levels, C-reactive protein, and white blood cell count	Negative association for *CPT1A* positive association for *ABCG1* & *SREBF1*	Good
			Replication set (KORA F3 study)	n = 499 (52%)	52.9 ± 9.6	27.2 ± 4.5				
			Replication set (InCHIANTI study)	n = 472 (45%)	71.2 ± 16	27 ± 4.3
Guay et al. (2014) (11)	Canada	Cross-sectional	In women	n = 37	39.6 ± 2	24.7 ± 0.8	*ABCG1*-CPG3	Age, waist circumference, and fasting blood TG	Positive association in women but no association in men	Fair
			In men	n = 61	46.3 ± 1.7	27.3 ± 0.5				
Peng et al. (2014) (13)	China	Cross-sectional	NA	n = 139 (64%)	NA	NA	*ABCG1*	Age, sex, smoking, lipid level, history of hypertension, and history of diabetes	No significant association	Good
Wei & Wu. (2018) (10)	USA	Cross-sectional	NA	n = 995	NA	NA	1, 7, 8, 10	Age, sex, study center, family relations	Negative association for *CPT1A* and positive association for *ABCG1*	Good
Sayols-Baixeras et al. (2016) (9)	Spain	Cohort	Discovery set	n = 645 (49%)	63.2 ± 11.7	26.9 ± 4.1	1, 2, 4, 5, 7, 12	age, gender, smoking exposure, batch effect, estimated cell count and surrogate variables	Negative association for *CPT1A* and positive association for *ABCG1* & *SREBF1*	Good
			Replication set	n = 2542 (46%)	66.3 ± 8.9	28.2 ± 5.4				
Irvin et al. (2014) (16)	USA	Cohort	Discovery set	n = 991 (48%)	48.8 ± 16	NA	7, 8, 9, 10	Age, gender, study site, cell purity, pedigree	Negative association Results of cg00574958 methylation were replicated, and same association observed	Good
			Replication set	n = 1261 (40%)	NA	NA				
Romanescu et al. (2018) (30)	USA	Cross-sectional	NA	n = 995	NA	NA	7, 8, 9, 10, 11	NA	Significant association	Good
Hedman et al. (2017) (14)	USA	Pooled analysis of Cohorts	Discovery set	n = 2306 (47%)	NA	NA	1, 2, 6, 7, 8, 9, 12, 14	Both discovery and replication sets, were analyzed in two models: – primary model: age, sex, white cell counts and batch effects – secondary model: primary model + BMI	Negative association for *CPT1A* and positive association for *ABCG1* & *SREBF1* - cg08129017 & cg01176028 were novel CpGs, had not found before	Good
			Replication set	n = 1955	NA	NA				
Gagnon et al. (2014) (31)	France	Cross-sectional	MARTHA study	n = 327 (21%)	44.1 ± 14.2	NA	7, 8	Age, gender, cell type, batch, and chip effects	Negative association	Fair
			F5L-pedigrees study	n = 199 (47%)	39.6 ± 16.9	NA				
Lai et al. (2016) (24)	USA	Cross-sectional	Discovery set	n = 653 (48%)	48 ± 16.2	28.2 ± 5.6	1, 7, 8, 9, 10, 12	age, sex, study site, baseline TG	Negative association between *CPT1A* and TG-AUC, and positive association between *ABCG1* & *SREBF1*, and TG-AUC * This study assesses the TG-AUC	Good
			Replication set	n = 326 (48%)	48.8 ± 16.9	28.3 ± 5.8				

NA: not available; N: number; TG: triglyceride; *ABCG1*: ATP-binding cassette sub-family G member-1 protein gene; *CPT1A*: carnitine palmitoytransferase-1A gene; *SREBF1:* sterol regulatory element-binding gene; CpGs: cytosine-guanine dinucleotides, BMI: body mass index, WHR: waist to hip ratio, MI: myocardial infarction; HbA1c: glycated hemoglobin A; PPL: postprandial lipemia; FH: familial hypercholesterolemia; AUC: area under the whole curve.

1 Values are mean ± SD (for age and BMI)

2 Methylation sites: *ABCG1*: cg06500161 (1), cg27243685 (2), cg07397296 (3), cg02370100 (4), cg01881899 (5), cg01176028 (6), *CPT1A*: cg00574958 (7), cg17058475 (8), cg09737197 (9), cg01082498 (10), cg26989316 (11), *SREBF1*: cg11024682 (12), cg20544516 (13), cg08129017 (14).

3 Quality Rating; we assess the quality of articles using the NIH checklist.

All the selected studies were conducted among individuals over 18 years of age, and their designs were cohort, cross-sectional and pooled analysis of cohorts. The sample tissue for all of them was blood cells. There was a wide difference in sample size. It varied from 37 to 4,261. Some studies conducted more than one assessment including discovery set, replication set and a pooled analysis of both. Methylation of CpG site in *ABCG1* gene (cg06500161, cg27243685, cg07397296, ABCG1-CPG3, cg01881899, cg02370100, cg01176028), *CPT1A* gene (cg00574958, cg17058475, cg09737197, cg01082498, cg26989316) and *SREBF1* gene (cg11024682, cg20544516, cg08129017) were assessed. Methylation at seven CpG sites in *ABCG1*, five CpG sites in *CPT1A* and three CpG sites in *SREBF1* genes showed significant association with TG.

### Association between DNA methylation of the *ABCG1* gene and serum TG levels

**Figure 2:**
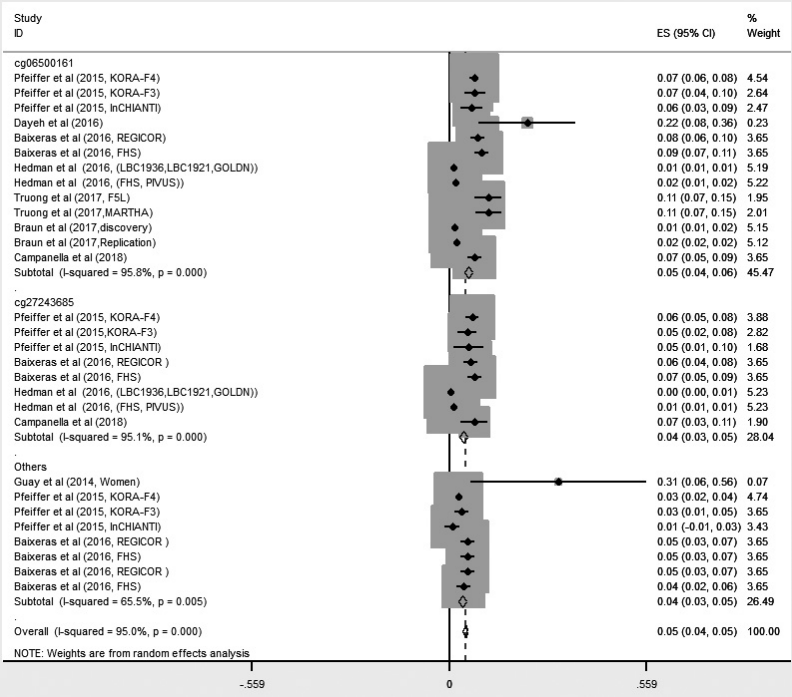
Associations of methylation of the *ABCG1* and triglycerides levels by CpG sites.

The findings of meta-analysis on 8 studies showed that DNA methylation of *ABCG1* gene had significant positive association with serum TG levels (β = 0.05; 95% CI [0.04, 0.05]) using random effect model. There was significant heterogeneity (P < 0.001), with I^2^ values of 95%. Therefore, the subgroup analysis, meta-regression and sensitivity analysis were used to explore the potential sources of heterogeneity (Figure 2).

### Subgroup analysis

Results of subgroup analysis based on CpG sites showed that DNA methylation of *ABCG1* gene in CpG site cg06500161 (β = 0.05; 95% CI [0.04, 0.06]; I^2^ = 95.8%), cg27243685 (β = 0.04; 95% CI [0.03, 0.05]; I^2^ = 95.1%) and others CpG sites (cg07397296, cg01881899, cg02370100, *ABCG1*-CpG3) (β = 0.04; 95% CI [0.03, 0.05]; I^2^ = 65.5%) was positively and significantly associated with serum TG levels. Only one paper had the relevant effect size for each of the following sites, cg07397296, cg01881899, cg02370100, *ABCG1*-CPG3. So we considered these sites as one group. The heterogeneity was significant for each three groups of CpGs (Figure 2).

### Meta regression

**Figure S1:**
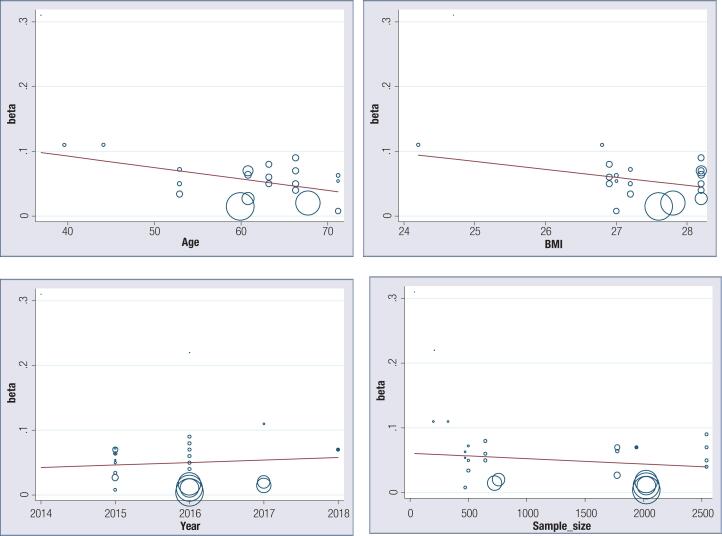
Meta-regression plot of the impact of age, BMI, year and sample size of studies on the association between *ABCG1* gene and TG levels, the size of the circles is depended on the weight of the study in the fitted random-effects meta-regression model.

**Figure S2:**
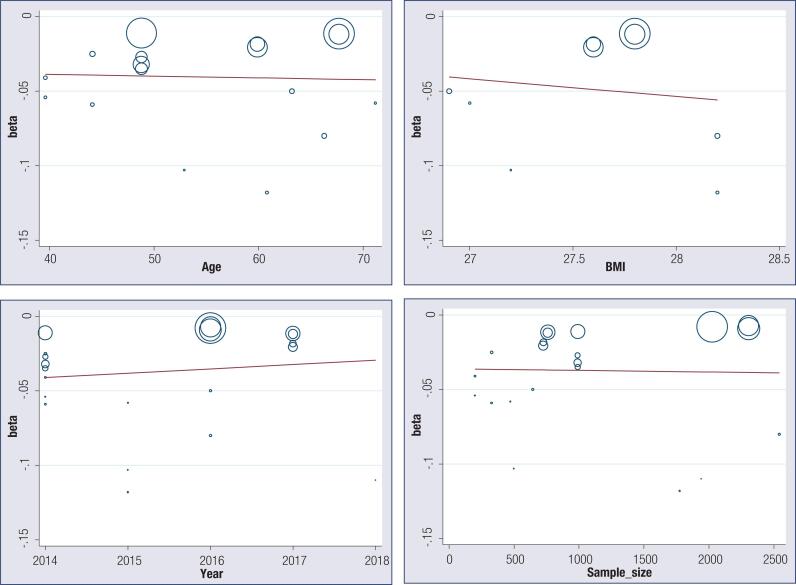
Meta-regression plot of the impact of age, BMI, year and sample size of studies on the association between *CPT1A* gene and TG levels, the size of the circles is depended on the weight of the study in the fitted random-effects meta-regression model.

**Figure S3:**
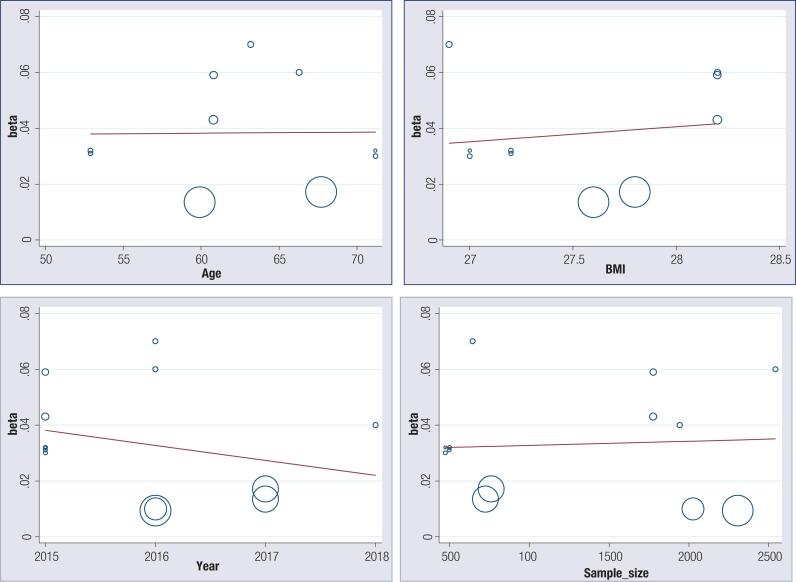
Meta-regression plot of the impact of age, BMI, year and sample size of studies on the association between *SREBF1* gene and TG levels, the size of the circles is depended on the weight of the study in the fitted random-effects meta-regression model.

Meta regression identified the mean age (β [standard error]: −0.002 [0.001]; p = 0.029) as the main source of the heterogeneity (R^2^ = 12.78%). It explained 92.65% of the heterogeneity. Mean BMI, sample size and the publication year of studies did not lead to heterogeneity (Supplementary file 2; figures S1, S2, and S3).

### Sensitivity analysis

Results of sensitivity analysis showed that the pooled effect size (β) and heterogeneity did not influence after excluding studies one by one. After excluding the study of Guay and cols. (11), which was conducted among women, the pooled effect size for studies did not change significantly (β = 0.045 [95% CI: 0.038, 0.052]; I^2^ = 95.2%). Furthermore, with excluding the study with unadjusted effect size (19), the pooled effect size for studies did not change significantly (β = 0.045 [95% CI: 0.038, 0.051]; I^2^ = 95.1%).

### Publication bias

Funnel plot showed asymmetry. The P-value for Egger's test was < 0.0001, which revealed obvious publication bias among studies. Therefore, trim and fill analysis was performed and the pooled effect size was obtained (β = 0.033 (95% CI: 0.026, 0.039); number of trimmed studies: 10).

### Association between DNA methylation of the *CPT1A* gene and serum TG levels

**Figure 3:**
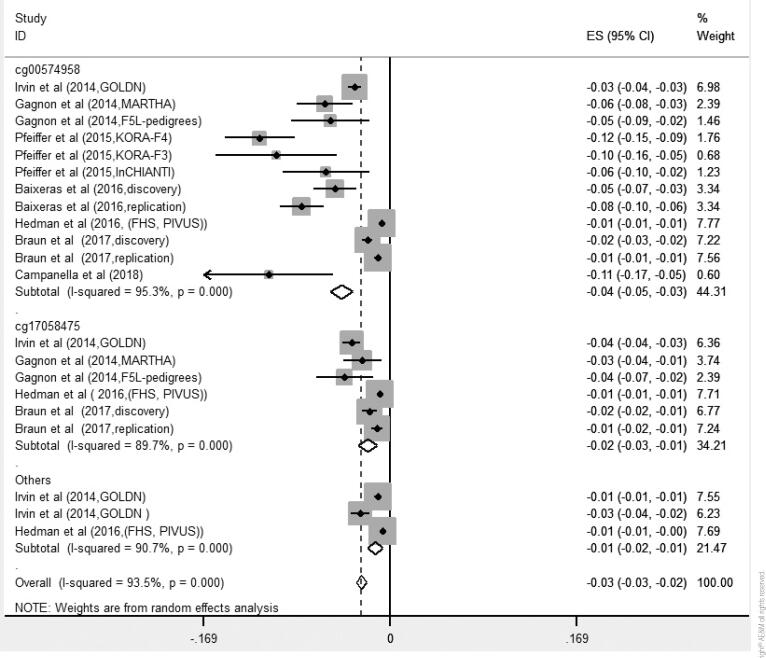
Associations of methylation of the *CPT1A* and triglycerides levels by CpG sites.

Results of meta-analysis on 7 studies indicated that the DNA methylation of *CPT1A* gene had significant inverse association with serum TG levels (β = −0.03 [95% CI: −0.03, −0.02]) using the random effects model. The heterogeneity was significant (I^2^ = 93.5%; P < 0.001). Figure 3 shows subgroup analysis based on CpG sites. The pooled estimates (β's) were negative and significant for CpGs of cg00574958 (β = −0.04; 95% CI [−0.05, −0.03]; I^2^ = 95.3%), cg17058475 (β = −0.02; 95% CI [−0.03, −0.01]; I^2^ = 89.7%) and other CpG sites (cg01082498, cg09737197) (β = −0.01; 95% CI [−0.02,−0.01]; I^2^ = 90.7%). Two studies had the relevant effect size for cg09737197, and one study had the relevant effect size for cg01082498, so we considered these sites as one group. The heterogeneity was significant for each three groups of CpGs (p < 0.001). The results of meta-regression analysis showed that mean age, mean BMI, sample size and the year of publication had no significant effect on the association between DNA methylation of *CPT1A* gene and serum TG levels (p > 0.05) (Figure 3).

Results of sensitivity analysis showed that the pooled effect size (β) was not influenced after excluding studies one by one. Funnel plot was asymmetry. The P-value for Egger's test was < 0.001, thus there was obvious publication bias among these studies. Trim and fill analysis were applied, but no studies were filled. It showed that the publication bias had a non-significant effect on the results.

### Association between DNA methylation of the *SREBF1* gene and serum TG levels

**Figure 4:**
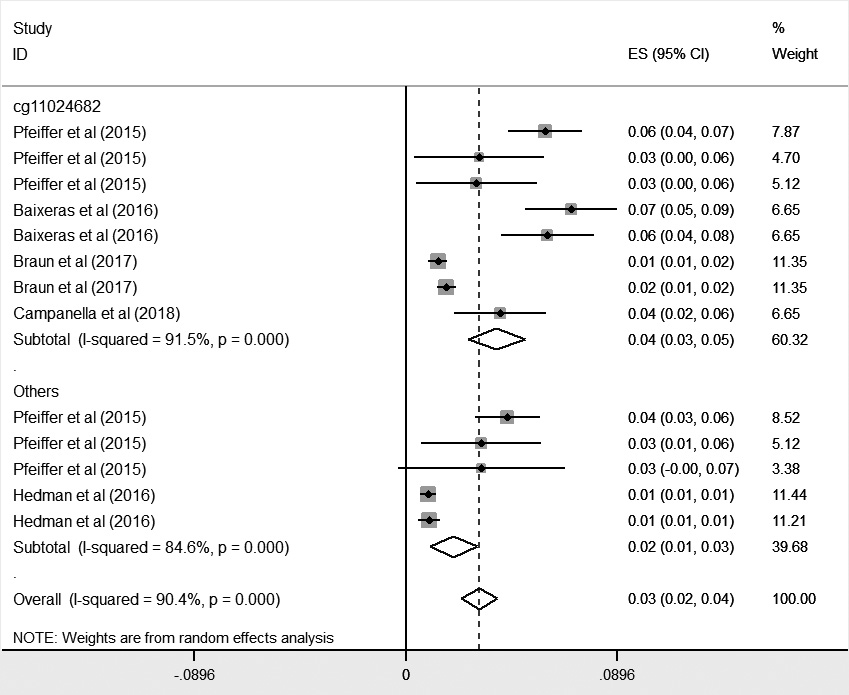
Associations of methylation of the *SREBF1* and triglycerides levels by CpG sites.

The findings of meta-analysis on 5 studies showed that DNA methylation of *SREBF1* gene was positively and significantly associated with serum TG levels (β = 0.03; 95% CI [0.02, 0.04]). The heterogeneity was significant (I^2^ = 90.4%; P < 0.001). The subgroup analysis based on CpG sites showed that pooled estimates for CpGs of cg11024682 (β = 0.04; 95% CI [0.03, 0.05, I^2^ = 91.5%]) and also for other group (cg20544516, cg08129017) (β = 0.02; 95% CI [0.01, 0.03, I^2^ = 84.6%]) were positive and significant (Figure 4). One study had the relevant effect size for cg20544516, and one study had the relevant effect size for cg08129017. So, we considered these sites as one group. The heterogeneity was significant for both groups. The results of meta-regression analysis indicated that mean age, mean BMI, sample size and year of publication had no significant association with the effect of *SREBF1* gene on serum TG levels (p > 0.05). Results of sensitivity analysis showed that the pooled effect size (β) was not influenced after dropping studies one by one. Funnel plot was asymmetry. The P-value for Egger's test was 0.001. Therefore, there was publication bias among these studies. So, trim and fill analysis was performed and the pooled effect size obtained (β = 0.015 [95% CI: 0.007, 0.023]); number of trimmed studies: 6).

## DISCUSSION

The present meta-analysis investigated the association between DNA methylation of different CpG sites of *ABCG1*, *CPT1A*, *SREBF1* and serum TG levels. Higher methylation of *ABCG1* and *SREBF1* genes, and lower methylation of *CPT1A* gene were significantly associated with higher serum TG levels. These findings can explain the reason of the large variations in the prevalence of hypertriglyceridemia in different populations.

### DNA methylation of *ABCG1* gene

*ABCG1* gene is located on chromosome 21 and encodes ATP-binding cassette sub-family G member-1 protein and controls macrophage lipoprotein lipase (LPL) activity. LPL controls the level of circulating blood lipids such as TG. Therefore, circulating TG could be affected by the level of *ABCG1* expression (20–22). However, there is no finding about a direct effect of *ABCG1* gene on TG level (15). Studies showed a negative association between *ABCG1* methylation and its expression (14,15,23). Several studies indicated significant association between the methylation level at different sites of *ABCG1* gene including cg06500161 (1,9,10,12,14,15,19,23–25), cg27243685 (9,14,15,23), cg07397296 (15), ABCG1-CPG3 (11), cg01881899 (9), cg02370100 (9), cg01176028 (14) and circulating TG levels. Different individual including healthy people (15,19), patients with familial hypercholesterolemia (11) and patients with venous thromboembolism (1) were included in studies, and this might be an underlying factor of diverse findings in different studies.

The current meta-analysis showed that methylation in cg06500161 and cg27243685 sites has greater impact on serum TG levels. Most of the included studies revealed significant positive association between *ABCG1* methylation and serum TG levels (1,9,12,14,15,19,23). While, one study on 139 individuals with coronary heart disease in China, did not show any significant association between *ABCG1* methylation and serum TG level (13). Another study on patients with familial hypercholesterolemia found significant association between *ABCG1* DNA methylation and HDL-C, LDL-C and TG levels only in women (11).

Study on 3296 participants from six cohorts in the Netherland suggested that the levels of blood TG influenced on the *ABCG1* methylation. They did not observe any causal effects of DNA methylation on lipid profiles (26). Studies demonstrated an association between higher *ABCG1* methylation and an increased risk of coronary heart disease, type 2 diabetes, and metabolic syndrome (13,15,19,27). Two population-based cohorts on 2306 individuals indicated that higher methylation at cg27243685 site of *ABCG1* gene increased the serum TG level and was associated with an increased risk of coronary heart disease (14).

### DNA methylation of *CPT1A* gene

Fatty acid oxidation in mitochondria provides an essential source of energy. A large portion of dietary fatty acid consists of long-chain fatty acids. However, they cannot simply enter to mitochondria and require special proteins to transfer them through the outer and inner mitochondria membrane. Carnitine palmitoytransferase-1 (*CPT1*) and carnitine palmitoytransferase-2 (*CPT2*) transfer long-chain fatty acids into mitochondria. *CPT2* exists in all tissues. However, *CPT1* is expressed mostly in three specific tissues including liver (*CPT1A*), muscle (*CPT1B*) and brain (*CPT1C*) (22,28). *CPT1A* gene, which located on chromosome 11, encodes *CPT1A* protein mostly in liver and some tissues such as heart, pancreas, endothelium, lung, skeletal muscle, and kidney (29). The level of methylation at five sites of *CPT1A* gene including cg00574958 (9,10,12,14–16,23–25,30,31), cg17058475 (10,12,14,16,24,30,31), cg09737197 (14,16,24,30), cg01082498 (10,16,24,30) and cg26989316 (30) had significant and inverse relationship with serum TG levels. Increasing the level of *CPT1A* methylation leads to decrease its expression. Thus increase the expression of *CPT1A* causes hypertriglyceridemia (12,16). DNA methylation mechanisms are reversible and new therapeutic approaches for treatment of hypertriglyceridemia can be used through modulating *CPT1A* expression (31). According to the present meta-analysis, methylation of cg00574958 and cg17058475 affects circulating TG levels more than other sites.

Dekkers and cols. suggested that blood TG level had causal effect on the level of *CPT1A* methylation but not vice versa (26). Another study on 992 participants in USA showed that both *CPT1A* methylation and blood TG levels might have reciprocal causal effect. However, because of the small sample size and lack of strong genetic instruments, they could not determine the direction association between *CPT1A* methylation and serum TG levels (25). It is suggested that *CPT1A* expression might act as a useful biomarker for CVD (28).

### DNA methylation of *SREBF1* gene

*SREBF1* (sterol regulatory element-binding transcription factor 1) gene is located on chromosome 17 and encodes sterol regulatory element binding proteins (SREBPs) (22). SREBP-1a and SREBP-1c are two isoforms of SREB proteins that are encoded by *SRBF1* gene. SREBF-1a modulates the expression of cholesterol and fatty acid biosynthesis genes, andSREBF-1c regulates the expression of fatty acid, phospholipid, and TG biosynthetic genes. Studies demonstrated that variation in lipid profiles could be related to these proteins (32,33). According to the current literature, the level of methylation at three loci of *SREBF1* gene including: cg11024682 (9,12,14,15,23–25), cg20544516 (15) and cg08129017 (14) has significant and positive association with serum TG level. There is an inverse association between *SREBF1* methylation and its expression. Thus, decreasing the expression of *SREBF1* might be the reason of hypertriglyceridemia (9,23). The current meta-analysis shows the effect of cg11024682 on serum TG levels is greater than other sites of *SREBF1*. Likewise, a study on 1,408 men and 1888 women of six cohorts showed that higher serum TG levels was correlated with higher methylation of cg11024682 (26). MicroRNA33b (MIR33b) is encoded by cg20544516 site on *SREBF1* gene. MIR33b suppress several genes such as *ABCG1* and *CPT1A* that they contribute to oxidation and transport of fatty acid (15,34,35).

Recent studies demonstrated that the methylation of *ABCG1*, *CPT1A*, and *SREBF1* genes was associated with some cardio-metabolic risk factors including total cholesterol and insulin levels, as well as anthropometric indices of general and abdominal obesity. Therefore, study on these genes can be important in prevention of non-communicable diseases (12,15,23).

According to the current findings, DNA methylation affects gene expression and new therapeutic pathways could be appeared for management of hypertriglyceridemia. Some medications including fenofibrate might affect serum TG levels through altering DNA methylation (36). However, a study with short-time follow up could not confirm these findings (37).

The present study has some limitations. First, the selection bias was unavoidable. Second, heterogeneity existed in our meta-analysis, and it could not be solved by applying any statistical method, differences in the genetic methods, age, BMI, or race of participants in various studies might be the reasons of this heterogeneity. The main strength of our study is its novelty; to the best of our knowledge, no previous systematic reviews and meta-analysis have specifically evaluated the relationship between methylation of different CpGs of some genes and serum TG levels. Meta-regression analysis was used to find potential variables as the main source of the heterogeneity.

In conclusion, the results of the present meta-analysis show that methylation of *ABCG1* and *SREBF1* genes have positive association with serum TG levels, whereas this association is inverse for methylation of *CPT1A* gene. DNA methylation on cg06500161 and cg27243685 at ABCG1 gene, cg00574958 and cg17058475 at *CPT1A* gene and cg11024682 at *SREBF1* gene are correlated with serum TG levels more than other CpGs. Although there are several studies that assessed the effect of DNA methylation on serum TG levels, but limited experience exists on the impact of serum TG levels on DNA methylation. Further studies with long follow up periods are needed to assess the causal effect of DNA methylation on serum TG levels and vice versa.

The role of epigenetic factors should be considered as one of underlying factors for the considerable variations in the prevalence of hypertriglyceridemia among different populations.

## SUPPLEMENTARY FILE 1

Search strategy:

A comprehensive systematic literature search was conducted in Medline database (PubMed), Scopus, and Cochrane library up end of July 2020, and we daily checked databases for any new publications up to end of 2020. We used the following search line for Medline database, and 632 papers were found:

(Triglyceride[Title/Abstract] OR TG[Title/Abstract] OR Triacylglycerol[Title/Abstract] OR Hypertriglyceridemia[Title/Abstract]) AND (Epigenetic[Title/Abstract] OR Epigenomic[Title/Abstract] OR methylation[Title/Abstract] OR “DNA methylation”[Title/Abstract] OR acetylation[Title/Abstract] OR “DNA acetylation”[Title/Abstract])

We searched the Scopus database and Cochrane library within Title, Abstract, and Keywords, using the following search string:

(Triglyceride OR Triacylglycerol OR Hypertriglyceridemia) AND (Epigenetic OR Epigenomic OR methylation OR “DNA methylation” OR acetylation OR “DNA acetylation”)

We found 1937 and 221 papers in Scopus and Cochrane library, respectively.

## References

[1] Truong V, Huang S, Dennis J, Lemire M, Zwingerman N, Aïssi D (2017). Blood triglyceride levels are associated with DNA methylation at the serine metabolism gene PHGDH. Sci Rep.

[2] Do R, Willer CJ, Schmidt EM, Sengupta S, Gao C, Peloso GM (2013). Common variants associated with plasma triglycerides and risk for coronary artery disease. Nat Genet.

[3] Sarwar N, Danesh J, Eiriksdottir G, Sigurdsson G, Wareham N, Bingham S (2007). Triglycerides and the risk of coronary heart disease: 10,158 incident cases among 262,525 participants in 29 Western prospective studies. Circulation.

[4] Reiner Ž (2017). Hypertriglyceridaemia and risk of coronary artery disease. Nat Rev Cardiol.

[5] Parhofer KG, Laufs U (2019). The Diagnosis and Treatment of Hypertriglyceridemia. Dtsch Arztebl Int.

[6] Ruiz-García A, Arranz-Martínez E, López-Uriarte B, Rivera-Teijido M, Palacios-Martínez D, Dávila-Blázquez GM (2020). Prevalence of hypertriglyceridemia in adults and related cardiometabolic factors. SIMETAP-HTG study. Clin Investig Arterioscler.

[7] Shin D, Kongpakpaisarn K, Bohra C (2018). Trends in the prevalence of metabolic syndrome and its components in the United States 2007-2014. Int J Cardiol.

[8] Gupta R, Rao RS, Misra A, Sharma SK (2017). Recent trends in epidemiology of dyslipidemias in India. Indian Heart J.

[9] Sayols-Baixeras S, Subirana I, Lluis-Ganella C, Civeira F, Roquer J, Do AN (2016). Identification and validation of seven new loci showing differential DNA methylation related to serum lipid profile: an epigenome-wide approach. The REGICOR study. Hum Mol Genet.

[10] Wei R, Wu Y (2018). Modification effect of fenofibrate therapy, a longitudinal epigenomic-wide methylation study of triglycerides levels in the GOLDN study. BMC Genet.

[11] Guay SP, Brisson D, Lamarche B, Gaudet D, Bouchard L (2014). Epipolymorphisms within lipoprotein genes contribute independently to plasma lipid levels in familial hypercholesterolemia. Epigenetics.

[12] Braun KVE, Dhana K, de Vries PS, Voortman T, van Meurs JBJ, Uitterlinden AG (2017). Epigenome-wide association study (EWAS) on lipids: the Rotterdam Study. Clin Epigenetics.

[13] Peng P, Wang L, Yang X, Huang X, Ba Y, Chen X (2014). A preliminary study of the relationship between promoter methylation of the ABCG1, GALNT2 and HMGCR genes and coronary heart disease. PLoS One.

[14] Hedman ÅK, Mendelson MM, Marioni RE, Gustafsson S, Joehanes R, Irvin MR (2017). Epigenetic Patterns in Blood Associated With Lipid Traits Predict Incident Coronary Heart Disease Events and Are Enriched for Results From Genome-Wide Association Studies. Circ Cardiovasc Genet.

[15] Pfeiffer L, Wahl S, Pilling LC, Reischl E, Sandling JK, Kunze S (2015). DNA methylation of lipid-related genes affects blood lipid levels. Circ Cardiovasc Genet.

[16] Irvin MR, Zhi D, Joehanes R, Mendelson M, Aslibekyan S, Claas SA (2014). Epigenome-wide association study of fasting blood lipids in the Genetics of Lipid-lowering Drugs and Diet Network study. Circulation.

[17] Page MJ, McKenzie JE, Bossuyt PM, Boutron I, Hoffmann TC, Mulrow CD (2021). The PRISMA 2020 statement: an updated guideline for reporting systematic reviews. BMJ.

[18] Study Quality Assessment Tools NHLBI, NIH [Internet].

[19] Dayeh T, Tuomi T, Almgren P, Perfilyev A, Jansson PA, de Mello VD (2016). DNA methylation of loci within ABCG1 and PHOSPHO1 in blood DNA is associated with future type 2 diabetes risk. Epigenetics.

[20] Frisdal E, Le Lay S, Hooton H, Poupel L, Olivier M, Alili R (2015). Adipocyte ATP-binding cassette G1 promotes triglyceride storage, fat mass growth, and human obesity. Diabetes.

[21] Hardy LM, Frisdal E, Le Goff W (2017). Critical Role of the Human ATP-Binding Cassette G1 Transporter in Cardiometabolic Diseases. Int J Mol Sci.

[22] Braun KVE, Voortman T, Dhana K, Troup J, Bramer WM, Troup J (2016). The role of DNA methylation in dyslipidaemia: A systematic review. Prog Lipid Res.

[23] Campanella G, Gunter MJ, Polidoro S, Krogh V, Palli D, Panico S (2018). Epigenome-wide association study of adiposity and future risk of obesity-related diseases. Int J Obes (Lond).

[24] Lai CQ, Wojczynski MK, Parnell LD, Hidalgo BA, Irvin MR, Aslibekyan S (2016). Epigenome-wide association study of triglyceride postprandial responses to a high-fat dietary challenge. J Lipid Res.

[25] Sayols-Baixeras S, Tiwari HK, Aslibekyan SW (2018). Disentangling associations between DNA methylation and blood lipids: a Mendelian randomization approach. BMC Proc.

[26] Dekkers KF, van Iterson M, Slieker RC, Moed MH, Bonder MJ, van Galen M (2016). Blood lipids influence DNA methylation in circulating cells. Genome Biol.

[27] Lira do Amaral C, Curi R, Caterina RD, Martinez JA, Kohlmeier M (2020). Principles of Nutrigenetics and Nutrigenomics [Internet].

[28] Irvin MR, Aslibekyan S, Hidalgo B, Arnett D (2014). CPT1A: the future of heart disease detection and personalized medicine?. Clin Lipidol.

[29] Schlaepfer IR, Joshi M (2020). CPT1A-mediated Fat Oxidation, Mechanisms, and Therapeutic Potential. Endocrinology.

[30] Romanescu RG, Espin-Garcia O, Ma J, Bull SB (2018). Integrating epigenetic, genetic, and phenotypic data to uncover gene-region associations with triglycerides in the GOLDN study. BMC Proc.

[31] Gagnon F, Aïssi D, Carrié A, Morange PE, Trégouët DA (2014). Robust validation of methylation levels association at CPT1A locus with lipid plasma levels. J Lipid Res.

[32] Vargas-Alarcon G, Gonzalez-Pacheco H, Perez-Mendez O, Posadas-Sanchez R, Cardoso-Saldaña G, Ramirez-Bello J (2019). SREBF1c and SREBF2 gene polymorphisms are associated with acute coronary syndrome and blood lipid levels in Mexican population. PLoS One.

[33] Liu JX, Liu J, Li PQ, Xie XD, Guo Q, Tian LM (2008). Association of sterol regulatory element-binding protein-1c gene polymorphism with type 2 diabetes mellitus, insulin resistance and blood lipid levels in Chinese population. Diabetes Res Clin Pract.

[34] Dávalos A, Goedeke L, Smibert P, Ramírez CM, Warrier NP, Andreo U (2011). miR-33a/b contribute to the regulation of fatty acid metabolism and insulin signaling. Proc Natl Acad Sci U S A.

[35] Rayner KJ, Fernandez-Hernando C, Moore KJ (2012). MicroRNAs regulating lipid metabolism in atherogenesis. Thromb Haemost.

[36] Yu JC, Hsu FC, Chiu YF (2018). Assessment of fenofibrate-methylation interactions on triglycerides using longitudinal family data. BMC Proc.

[37] Das M, Irvin MR, Sha J, Aslibekyan S, Hidalgo B, Perry RT (2015). Lipid changes due to fenofibrate treatment are not associated with changes in DNA methylation patterns in the GOLDN study. Front Genet.

